# Missing channels in two-colour microarray experiments: Combining single-channel and two-channel data

**DOI:** 10.1186/1471-2105-8-26

**Published:** 2007-01-25

**Authors:** Andy G Lynch, David E Neal, John D Kelly, Glyn J Burtt, Natalie P Thorne

**Affiliations:** 1 Department of Oncology, University of Cambridge, Cambridge, UK; 2 Department of Applied Mathematics and Theoretical Physics, University of Cambridge, Cambridge, UK

## Abstract

**Background:**

There are mechanisms, notably ozone degradation, that can damage a single channel of two-channel microarray experiments. Resulting analyses therefore often choose between the unacceptable inclusion of poor quality data or the unpalatable exclusion of some (possibly a lot of) good quality data along with the bad. Two such approaches would be a single channel analysis using some of the data from all of the arrays, and an analysis of all of the data, but only from unaffected arrays. In this paper we examine a 'combined' approach to the analysis of such affected experiments that uses all of the unaffected data.

**Results:**

A simulation experiment shows that while a single channel analysis performs relatively well when the majority of arrays are affected, and excluding affected arrays performs relatively well when few arrays are affected (as would be expected in both cases), the combined approach out-performs both. There are benefits to actively estimating the key-parameter of the approach, but whether these compensate for the increased computational cost and complexity over just setting that parameter to take a fixed value is not clear. Inclusion of ozone-affected data results in poor performance, with a clear spatial effect in the damage being apparent.

**Conclusion:**

There is no need to exclude unaffected data in order to remove those which are damaged. The combined approach discussed here is shown to out-perform more usual approaches, although it seems that if the damage is limited to very few arrays, or extends to very nearly all, then the benefits will be limited. In other circumstances though, large improvements in performance can be achieved by adopting such an approach.

## Background

Cyanine-5 (Cy5) and cyanine-3 (Cy3) are popular dyes in use for microarray experiments. The destructive effect of ozone on the Cy5 dye, in particular, has been discussed [1,2], and a mechanism for the effect reported (see for example the poster Garner J, Howerton K, Schwalm J and Getts R "Development of a Protective Coating Designed to Preserve the Absorption and Emission Properties of Flourescent Dyes Used on Microarrays" currently available from [3]). The consequence of this phenomenon is that for two-channel microarray experiments, conducted without protection in a high-ozone environment, one channel of information may be partially or completely corrupted.

While this relationship may not have been accepted universally [4], the fact that arrays with a defective Cy5 channel exist is not disputed. The advice from a producer of microarray systems [5] is either to avoid high ozone levels when conducting experiments (passively or actively), or to use one of a number of Cy5 stabilizing solutions that have been shown to prevent a good deal of the degradation.

It is indeed ideal to avoid this problem, however it remains the case that some experiments have suffered and will suffer in this way. The task is then to conduct a valid and informative analysis. Options include discarding the arrays that have problems in their Cy5 data, or discarding the Cy5 data from all arrays and performing a Single Channel analysis. Further options are to ignore the problem (and presumably either display suitable caution in the interpretation or trust that the effect is regular enough that it can be normalized away), or to abandon the data entirely and repeat the experiment. One can imagine circumstances where all of these approaches might suffice, however one can also see that there are drawbacks to each.

In this paper we are motivated by a dataset where the Cy5 channel has been corrupted for a number of arrays. We comment initially on whether this may have been due to ozone, but focus on an approach to their analysis regardless of the cause of the problem (although with the assumption that this is a non-trivial disruption to the Cy5 channel). We further show that this approach produces sensible and consistent results, without incurring the problems of the approaches described above. These results will be substantiated by a large simulation experiment that we also present.

The motivating dataset consists of 187 microarrays from two experiments. One was conducted at times of generally high ozone and the other at times of generally low ozone. The arrays represent samples from 121 individuals and have a common reference in the Cy5 channel, which also appears in the Cy3 channel for two of the arrays. Details are given in Table 1.

**Table 1:** Characteristics of the study samples

	Experiment 1	Experiment 2	Combined
Ozone	High	Low	

Arrays			
Number	90	97	187
Cancer/Normal/Reference	74/14/2	86/11/0	160/25/2
			
Patients			
Number	79	90	121
Cancer/Normal/Both	65/11/3	79/10/1	99/14/8
Providing ≥2 Cancer Arrays	6	6	47
Providing ≥2 Normal Arrays	0	0	3
Providing Normal and Cancer Arrays	4	1	8

Detailing the number of normal and cancer tissue arrays used in the study, the numbers of patients who have provided normal or cancer arrays (or both), and the number of patients that feature on more than one array. Recall that all arrays have the Reference in the Cy5 channel.

**Figure 1:**
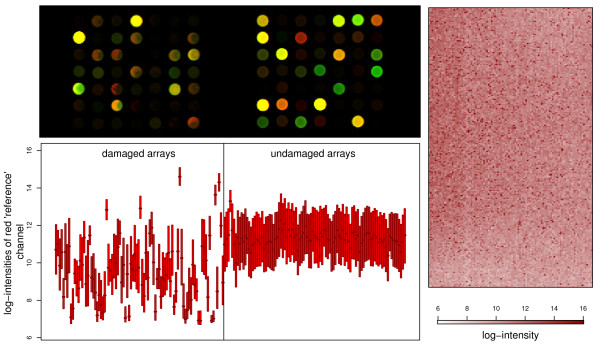
**Evidence of the effect of ozone**. (Top left) Depicting the spot morphology typically observed, first in ozone-affected arrays and then in undamaged arrays. Note the greater homogeneity of spot arising from the undamaged arrays when contrasted with the 'phases of the moon' demonstrated by the damaged arrays. (Bottom left) Depicting the log-intensities observed in the red foreground channels of first ozone-affected arrays and then undamaged arrays. Note that the damaged arrays show much greater inter-array variation, generally lower intensities and often much lower intra-array variance. (Right) A representation of the red channel from a particular ozone-affected array. Missing values (as are part of the design of the platform) have been replaced by an average of their neighbours' values to avoid distraction. Note the lower intensities in the upper, lower and right-hand sides, and the still-observable zig-zag of control spots approximately a quarter of the way in from the left-hand side.

Evidence of the effects of ozone can be illustrated both by examination of an affected array at an individual spot level and across the array in its entirety (Figure 1). Affected spots appear less homogenous in colour, with many showing 'phases of the moon' while others exhibit a doughnut-like pattern. The 'visible' gradient of the red signal away from an edge or corner, as visible in a number of arrays, suggests an environmental factor. The difference in gradients between affected and unaffected arrays allows for fair discrimination (not shown) between ozone-affected and unaffected arrays. Although direct ozone measurements are not available, the principal ozone monitoring site for Cambridgeshire is a little over ten miles from the site of the microarray experiment and should give some indication of the relative levels of ozone.

**Figure 2:**
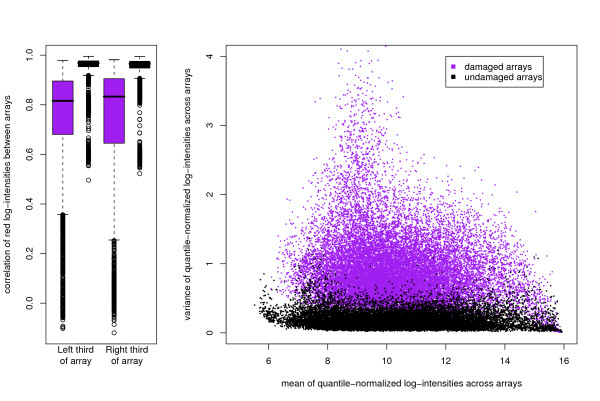
**The extent of the damage caused by ozone**. (Left) The spearman's rank correlation of log-intensities between arrays. The correlation between the log-intensities on the left 35 columns of the array on one microarray and another microarray is calculated for each pair of ozone-damaged arrays. These correlations are depicted in the boxplot. This is repeated for the undamaged arrays and also for the right 35 columns for both the damaged and undamaged arrays. Note that the exhaustive pairwise comparisons are not independent, but the plot illustrates the amount of information lost. (Right) Illustrating the increased variance associated with spots on ozone-affected arrays. After quantile normalization of the entire set of red-channel log-intensities, the mean and variance of observations of each spot were calculated firstly across the damaged arrays and secondly across the undamaged arrays. Note that the range of mean-intensities observed is lower in the damaged arrays.

The profiles of intensities of the red channel are clearly more variable (and generally lower) for the duration of the earlier ozone-affected arrays. The greater variability is emphasized in Figure 2, where even after normalization, greater variation in log-intensities is apparent. That figure also demonstrates the greater problem facing the ozone damaged data. The effects of the ozone are not something systematic, rather they can leave data that have no hope of being normalized as the message is lost. Indeed, within sub-regions of the arrays, the inter-array Spearman (rank) correlations can even achieve negative values. Six approaches to analysis are considered in this paper. Three of these are referred to as the 'Unaffected Data' analysis (i.e. discarding all affected arrays and analysing those that are left in a conventional manner), the Inclusive analysis (i.e. ignore the problem, include all arrays and analyse in a conventional manner), and the self-explanatory 'Single Channel' analysis of the Cy3 channels. The three new approaches are a combined analysis with the active estimation of a key parameter *k *(denoted the Combined(*k*_est_) analysis), and two combined analyses with *k *fixed to be 0.25 or 0.5 (denoted Combined(*k*_fix = 0.25_) and Combined(*k*_fix = 0.5_) respectively), the details of which are given in the methods section. Further materials, including the R computer code to perform these analyses, will be available to download [6].

In addition, for some specific comparisons, results are presented of a Single Channel analysis applied only to the Cy3 channel of arrays that have been affected by ozone. We would not suggest that this would ever be a sensible approach, but the approach provides a limit to the Combined(*k*_fix = X_) family of distributions as will be clarified at the appropriate juncture.

We focus on the task of distinguishing cancerous samples from normal (non-cancerous) samples through modelling of gene-expression levels. This is a well-understood problem, and allows for a comparison of the performance of the methods without distraction. We readily acknowledge that this may not be the most important biological comparison, but reaffirm that the purpose of this exercise is to compare the methods of analysis.

## Results

### Simulation experiment

**Figure 3:**
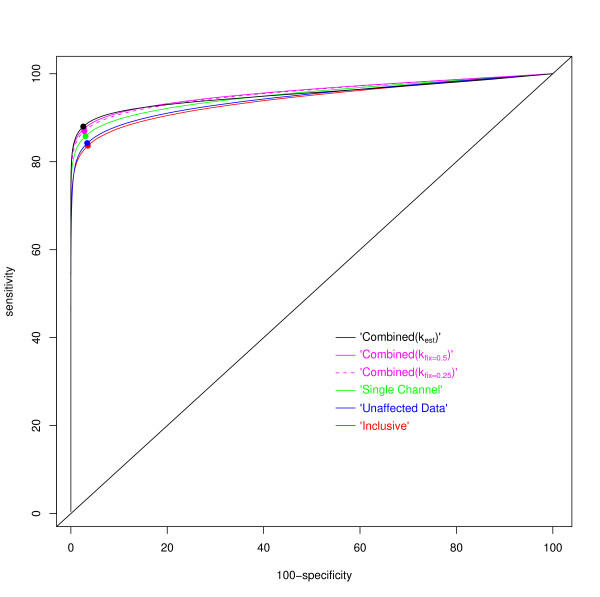
**Receiver Operating Characteristic curves depicting the performance of the methods in simulation**. For each of the six methods compared in the simulation experiment, the curves plot the percentage of the (in total) 400,000 simulated differentially expressed (d.e.) genes that would be returned in gene-lists of varying length (the sensitivity) against the percentage of the 1,857,500 simulated non-differentially expressed genes that are correctly not-returned in the same gene list (the specificity). Two trivial cases are the genelist of length zero (none of the d.e. genes are correctly returned, none of the non-d.e. genes are incorrectly returned = 0% sensitivity and 100% specificity) and that of length 22,575 (100% sensitivity and 0% specificity). The diagonal line indicates the performance that could be acheived by random classification. As a general rule, the nearer to the top left corner the curve passes, the better the method.

**Figure 4:**
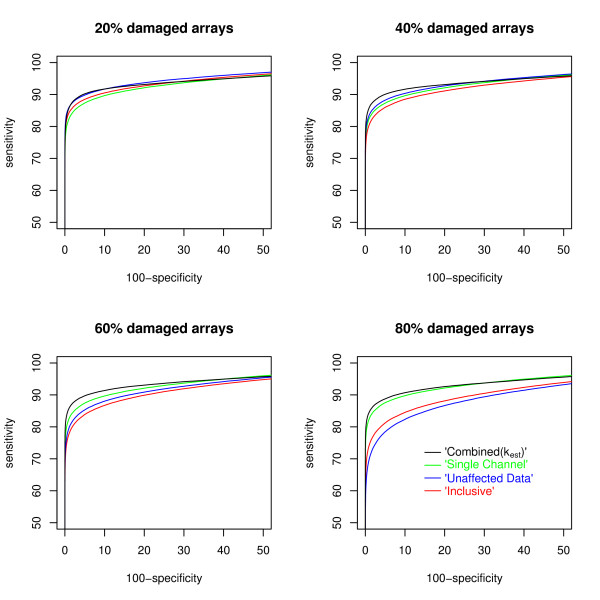
**Receiver Operating Characteristic curves depicting the performance of the methods in simulation by level of damage**. Depicting the sensitivity and specificity (dependent on choice of cut off) of four of the methods described here in terms of their ability to detect the differentially expressed genes under the four different levels of damage. The Combined(*k*_fix_) methods are omitted for sake of clarity, and the top left corner only is presented for the same reason. For an explanation of ROC curves see Figure 3.

From the 100 simulated datasets and analyses, summarized in Table 2, it is apparent that under these conditions the method presented in this paper shows an improvement on the other approaches in terms of identifying differentially expressed genes (Figures 3 and 4). Most of the trends are as would be expected; the Single Channel analysis is consistent across the four levels of damage to the data and the other methods show a decline in performance as the percentage of damaged arrays increases. Moreover, the differences between methods decrease as the level of damage decreases. Again this is a perfectly reasonable property. We should though note that the simulation study will be biased in two ways. Firstly, the spatial effects of the ozone damage have not been included, so the Inclusive approach will probably appear to do better than it should. Secondly, the values of *k *have been homogenized (if only a little) in the simulation as compared to the real data, thus the benefits of the Combined(*k*_est_) may be being downplayed.

**Table 2:** Performance of the five methods in simulation

	Method of Analysis					
Percentage arrays damaged	Single Channel	Unaffected Data	Inclusive	Combined (*k*_est_)	Combined (*k*_*fix *= 0.5_)	Combined (*k*_*fix *= 0.25_)

20	85,780	88,197	86,899	**88,393**	87,763	88,017
40	85,776	86,592	84,557	**88,296**	87,317	87,704
60	85,799	83,958	82,650	**88,054**	86,765	87,402
80	85,893	78,134	80,411	**87,252**	86,016	86,848
						
All Simulations	343,248	336,881	334,517	**351,995**	347,861	349,971

Presented are the numbers of differentially expressed genes that would be identified by taking a list of the top 4,000 genes in each case. Recall that there are indeed 4,000 differentially expressed genes, so the numbers are out of 100,000 (25 times 4,000) for the sub experiments and out of 400,000 (100 times 4,000) overall.

At all levels of damage, the Combined(*k*_est_) method presented within this paper performed best for detecting differentially expressed genes, while the Combined(*k*_fix_) approaches were generally close seconds. As an example of the advantage of the active estimation of *k*, when 80% of the arrays are damaged, the Combined(*k*_est_) approach correctly identifies 872 differentially expressed genes that are missed by the Combined(*k*_fix = 0.5_) approach, while only 468 are discordant in the other manner (See Additional file 1). With these values, McNemar's test returns a p-value of less than 0.0001. At high levels of damage, the Single Channel analysis approaches this level of performance, whilst at low levels the Unaffected Data analysis performs relatively well.

The Inclusive analysis surprisingly outperforms the Single Channel analysis when only 20% of arrays are damaged and outperforms the Unaffected data analysis when 80% of the arrays are damaged, but loses out in every other comparison (McNemar's test: *p *< 0.0001 in each case). In both of the cases when it shows superiority it has available 80 channels of undamaged information that the inferior method does not. That this is enough to overcome the 20 damaged arrays included in the victorious comparison with the Single Channel method is understandable, that it overcomes the 80 damaged arrays included in the victorious comparison with the Unaffected data approach is less so, and may reflect our failure to simulate spatial structure in the ozone damage, allowing the damage to average out over such a number of arrays. The Unaffected analysis outperforms the Single Channel approach at damage levels of 20% and 40% but is outperformed at the 60% and 80% levels (McNemar's test: *p *< 0.0001 in each case). The Combined(*k*_fix = 0.5_) approach outperforms the Combined(*k*_fix = 0.25_) approach at all levels of damage.

When the magnitude of the simulated effect size (i.e. the modelled log-ratio) is greater than 0.5, the stronger methods consistently correctly return 95% of the differentially expressed genes. The greatest interest for differentiation between methods then lies in those genes where the effect was simulated to be less than 0.5. Here, the Single Channel and Inclusive approaches perform far more poorly, with the Unaffected data approach doing very well at low damage levels, but not performing to the same standard at other damage levels.

Full details of the pairwise comparisons are presented in Additional file 1.

### Application to example data

The rankings of the top genes exhibiting differential expression between normal and cancer samples are listed in Additional file 2. Broadly speaking, the same genes are appearing towards the top of all of the lists. There are though distinctions to be made. The table was ordered by the results from the Combined(*k*_est_) analysis, and taking this as a reference one can see that the Combined(*k*_fix_) and Single Channel analyses perform most similarly.

**Figure 5:**
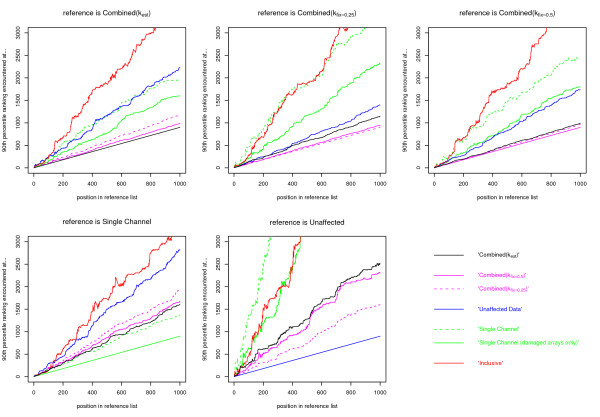
**Comparison of the similarity of the results of the methods of analysis**. Illustrating, for a reference genelist, how far down another list of genes one would have to go to include 90% of the first *x *genes returned by the reference, where *x *is depicted on the x-axis. The genelists depicted are those associated with the six different analyses of the real data. Additionally, an analysis of the single (green) channel only from the ozone affected arrays is included. Note that one line in each plot compares the reference method to itself and so naturally has the equation *y *= 0.9*x*.

Figure 5 depicts the distance down the list one would have to go to return 90% of the genes returned so far by the reference method. So by definition, when the reference is the Combined(*k*_est_) analysis, one needs to go down to position 90 of the Combined(*k*_est_) list to include 90% of the top 100 genes in that list, and down to position 900 to include 90% of the top 1,000. By contrast one has to go down approximately 2,000 genes in the list generated by the Unaffected data approach in order to include 90% of the top 1,000 genes returned by the Combined(*k*_est_) method. Additional information summarizing the comparisons is given in Table 3.

**Table 3:** Numerical comparison of analysis methods applied to the real data

	Comb. (*k*_*est*_)	Comb. (*k*_*fix *= 0.5_)	Comb. (*k*_*fix *= 0.25_)	Unaffected	Single Channel	SC (damaged)	Inclusive
Combined(*k*_est_)	-	0.88	0.89	0.83	0.70	0.62	0.57
Combined(*k*_fix = 0.5_)	904	-	0.98	0.80	0.78	0.71	0.56
Combined(*k*_fix = 0.25_)	842	922	-	0.88	0.74	0.66	0.57
Unaffected Data	669	723	788	-	0.58	0.48	0.53
Single Channel	750	725	686	575	-	0.84	0.56
SC (damaged arrays)	710	656	612	492	788	-	0.53
Inclusive	620	629	631	591	660	605	-

For every pair of analyses: Presented in the upper right corner is the Spearman's rank correlation comparing for all genes the two sets of log-odds of being differentially expressed. In the bottom left is given the number of genes common to the top 1,000 returned by the two methods.

**Figure 6:**
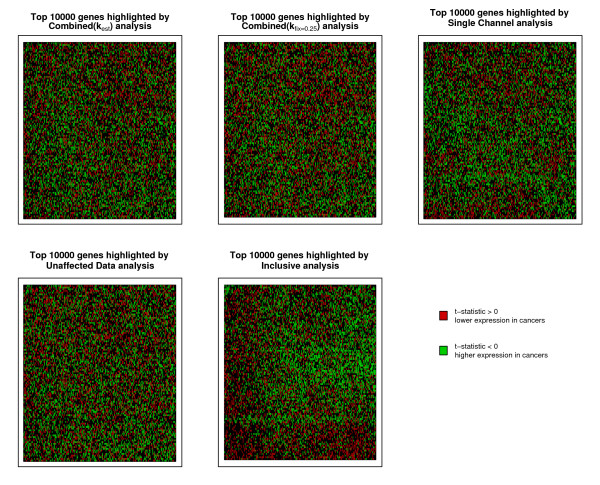
**The locations on the array of nominated genes**. Depicting the spatial effects of the results from applying the five methods on test to the motivating dataset. Taking the top 10,000 genes from the list of all genes ranked by the log-odds of their being differentially expressed, the direction of the differential expression is indicated by the colour.

It is apparent from Figure 5 that the results from the Combined(*k*_est_) and Combined(*k*_fix_) approaches are the most similar, as might be expected; in particular the Combined(*k*_fix = 0.5_) method. The Single Channel approach produces results that are closer to those from the Combined(*k*_est_) method than they are to those from the Combined(*k*_fix_) analysis, or indeed than the Combined(*k*_est_) analysis is to the Unaffected data analysis. One constant is that the Inclusive results are vastly different to all of the other methods, with only the Single Channel and Unaffected data results showing a difference of similar magnitude. It is interesting to note that the lines are not uniformly ordered as the position in the reference list increases, but can converge, diverge and cross. For example when the reference is one of the Combined approaches (especially the Combined(*k*_fix = 0.5_) method), the Single Channel (damaged arrays only) and Inclusive approaches start off remarkably similarly before diverging at some point. Taking the Single Channel as the reference, the Inclusive analysis outperforms the Unaffected data analysis until well after position 100 in the reference gene list, at which point it deteriorates in relative performance rapidly. The reason for the distinct nature of the Inclusive results may be apparent upon consideration of the spatial location on the array of returned genes. The locations of the top 10,000 genes from each method are depicted in Figure 6 coloured according to whether the detected change was up or down in the cancer samples. The locations from the Combined(*k*_est_), Combined(*k*_fix_), Single Channel and Unaffected analyses appear random, whereas there is a clear spatial effect in the results of the Inclusive analysis.

**Figure 7:**
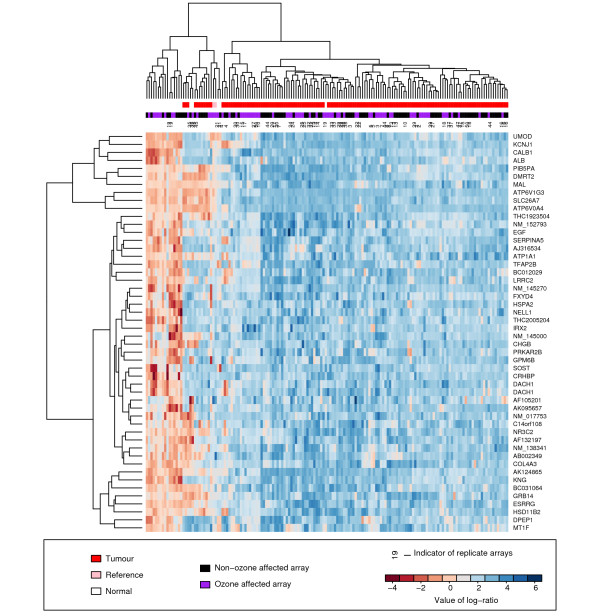
**Heatmap showing discrimination of normal and cancer samples using the top fifty genes identified by the Combined(*k*_est_) analysis**. A heatmap showing the clustering of arrays by genes chosen for their diagnostic ability. As well as the usual details are indicated the ozone-affected status of the arrays and details of which arrays were repeated in both the ozone-affected and unaffected experiments. Note that the ozone-affected status of the array has little bearing on the clustering.

A check that the Combined(*k*_est_) method is producing sensible results is given in the clustering and heatmap shown in Figure 7. Here fifty genes that showed most evidence of differential expression are plotted. There is then limited surprise that good discrimination is seen between the normal and cancer samples. However also indicated is whether each sample was from an ozone-affected array or not, and crucially pairs (or more) of arrays are indicated that are heterogeneous with respect to ozone damage but originate from the same biological sample.

It can be seen that there is no obvious tendency for the arrays to divide into ozone-affected and ozone-unaffected clusters. Some low-level grouping of the same ozone-effect type will occur by chance of course, and some further will be driven by pairs that are homogeneous and originate from the same sample (not indicated). So there really is no evidence of a noticeable effect here. Moreover, the replicates cluster together well, demonstrating that the loss of the Cy5 channel data is being overcome in a satisfactory manner.

### Estimation of *k*

**Figure 8:**
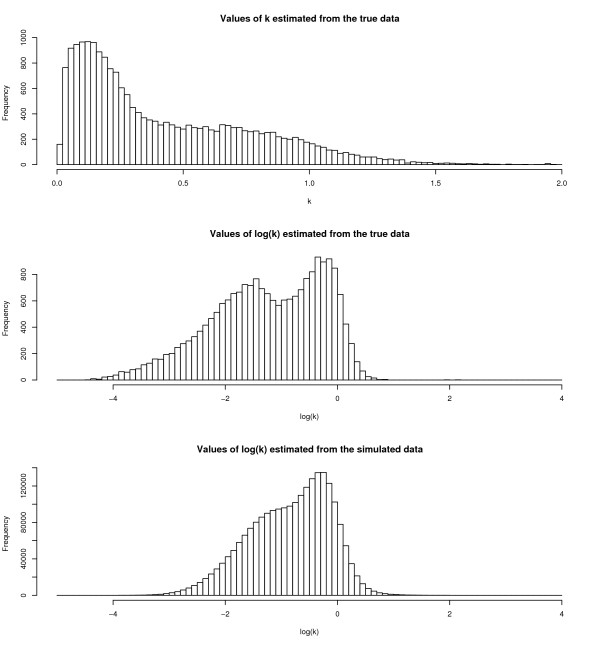
**Illustrating the distributions of *k *and log(*k*)**. Histograms showing the distribution of i) the 22,153 estimates of *k *obtained from the real data, ii) the 22,153 estimates of log(*k*) obtained from the real data, this being in many ways a more natural scale on which to consider *k*, and iii) the 2,257,700 estimates of log(*k*) obtained from the simulated data.

In Figure 8 are illustrated the values of *k *estimated when applying the Combined (*k*_est_) method to the example data. The distribution of the logarithm of *k *is also presented, as it is in the log-space that *k *is shrunk. While a normal approximation for log(*k*) is not fantastic, it is certainly better than that for *k*. The fixed estimates of 0.25 and 0.5 are appropriate for a *k *that has a mean of 0.43, geometric mean of 0.28, median of 0.31, and mode of 0.13.

Looking at Figure 5. If we note that the Unaffected data analysis is essentially a Combined(*k*_fix = 0_) approach, and the Single Channel analysis applied only to the damaged arrays is essentially a Combined(*k*_fix = ∞_) approach then we can see that, for example when the reference is Unaffected, the order of *k *= 0, *k *= 0.25, *k *= 0.5, *k *= ∞ is preserved in the ordering of the lines. The Combined(*k*_est_) approach slots in a little after the Combined(*k*_fix = 0.5_) method in this ordering. This might be taken as weak evidence in favour of a Combined(*k*_fix > 0.5_) approach, but observation of Figure 8 makes such a choice hard to countenance.

The values of log(*k*) produced from the simulation experiment are also presented in Figure 8. As might be anticipated, the mixing of components of the data to generate new datasets leads to some homogenization of the estimates of *k*. This is apparent despite the fact that the simulated datasets are smaller than the motivating dataset meaning that the individual estimates of *k *might be expected to be less precise and thus lead to a heavier-tailed distribution. Nevertheless, the distribution of log(*k*) estimated from the simulated data is recognisably of the form demonstrated by the example data, albeit that the two modes have somewhat converged.

## Discussion

There is a distinct spatial aspect to the damaged data. This is clear both from the initial presentation of the data (Figure 1) and the results from the Inclusive analysis. The simulated data did not include a spatial effect in the damage inflicted. Nevertheless, they (along with the analysis of our example data), demonstrated that the inclusion of those damaged data would be detrimental to the analysis.

It is no surprise either that the Single Channel analysis performs well regardless of the percentage of damaged arrays in the experiment as it does not use the channel that is being damaged. The other methods naturally improve as more arrays survive intact, because they have more reliable information available. Therefore it is inevitable that there will be a proportion of missing channels above which the Single Channel approach will do better than the Unaffected Data analysis and below which it will perform worse. That it appears to be at about the 50% mark in our simulations is a little more surprising. It is worthy of note that in those cases when the Single Channel approach does outperform the Unaffected Data analysis, the margin is somewhat greater than when the reverse is true.

The Unaffected Data analysis could be regarded as a Combined analysis with *k *set to zero. From this viewpoint, it is not surprising that the Combined(*k*_fix = 0.25_) approach lies between the Unaffected Data analysis and the Combined(*k*_fix = 0.5_) approaches in the results that it produces. If the Unaffected Data analysis has available about half of the arrays (as it does in our sample), and the variance of the log-ratio is approximately half of the variance of the log-expression levels used in the Single Channel analysis, then we might anticipate that the precision of the estimates from the two approaches (Unaffected Data and Single Channel) would be approximately equal to each other.

That the Single Channel and Unaffected Data approaches therefore differ so markedly in their results for the example data, despite our anticipating that their overall performances might be broadly similar, is evidence that a unifying approach such as the Combined analysis really does have something to offer. The results of the simulation experiment suggest that the increase in information that the Combined approach has available more than makes up for the extra complexity of the model. The successful pairing of replicates in the clustering shown in Figure 7 gives additional evidence of the value of this approach. In the simulation experiment, all of the methods are guaranteed high specificity due to the ratio of differentially expressed to non-differentially expressed genes. With 18,575 out of 22,575 simulated genes being non-differentially expressed, the formula for specificity will be at worst (18,575 - *x*)/18,575 where *x *is the number of genes nominated as being differentially expressed. So we would have to take a list of 1,858 genes as being differentially expressed before even the least specific of methods could drop below 90% specificity.

Complementary to this is the problem that differentially expressed genes differ in their level of differential expression. There will be some genes that, due to their large associated effect, even the least discriminatory methods of analysis would struggle to miss. A method of analysis may then appear to be competent in the ROC curve merely by harvesting the low-hanging fruit. Results that are so self-evident though are unlikely to be of great interest. It is in the detection of genes with a small differential expression that the improvements offered by the Combined(*k*_est_) approach are most apparent.

Our motivating example, and thus our simulated data, originates from an experiment where the affected channel was a common reference. This is clearly not always the case, and we can speculate on the effect that relaxing this condition might have. We will assume that we still have a situation where there are three groups (normal, cancer and reference), and we are only primarily interested in a comparison between the normal and cancer groups. In such circumstances, a Single Channel analysis will not make use of the reference data in the comparison of interest, save for using it to help model the residual variances.

It may be desirable to ignore entirely arrays providing only reference data in a Single Channel analysis, unless one is confident that the minimal extra information provided will outweigh the strong modelling assumptions being made both in the Single Channel normalization and the linear modelling itself when those arrays are included. A Combined analysis method on the other hand will actively use arrays providing information only on the reference group, provided there exist undamaged arrays within which a reference-to-normal or reference-to-cancer comparison is being made. Additionally of course, the Combined analysis is able to make use of normal and cancer samples on the unaffected channels of damaged arrays. Thus we would anticipate that the current experimental design is one where the Single Channel would perform at its best relative to the other methods, and that in other designs the advantages offered by a Combined approach would be even more convincing.

The values of 0.5 and 0.25 were obtained from experimental observations of two-channel cDNA microarrays produced at the Peter MacCallum Cancer Institute and the University of California Berkeley respectively. That the Agilent arrays appear to produce values of *k *that are not dissimilar is evidence towards some commonality across platforms. However, that *k *= 0.5 seems to perform better than *k *= 0.25 implies that the Single Channels contain more information relative to the log-ratios than anticipated from the cDNA microarrays. This is in keeping with the Agilent technologies' reputation for being particularly robust, and suggests that in fact there may be scope for a platform-dependent improved Bayesian shrinkage step in the active estimation of *k*.

## Conclusion

Ignoring the issue of damage to one of the channels results in poor results if such damage exists. Moreover the more damaged arrays there are, the worse the performance of such an approach. The problems are not only due to an increased variance resulting from the loss of information (as seen in the simulation), but also a spatial bias that is evident in the results. A Single Channel approach on the other hand may perform very well in suitable conditions (damage to a majority of arrays, and the lost channel not containing direct information about the contrast of interest), but is outperformed when only a small number of arrays are damaged, by an approach based on simply discarding those arrays. A complementary result is that an analysis of Unaffected arrays performs better the more undamaged arrays there are, but is less reliable than a Single Channel analysis if there are few undamaged arrays.

A Combined analysis has been shown here to offer improvements over the other analyses considered. It could be regarded as producing results that are a compromise between those from a Single Channel analysis and those from an Unaffected Data analysis, and in objective tests it outperforms both. The additional modelling assumptions and complexity are handsomely compensated for by the additional information obtained when including all of the available data. Indeed, if one is using a prescribed value of *k *in the analysis, then many of the rewards are obtained for a minimum of additional computational effort.

## Methods

### A 'Combined' approach to the linear modelling

Our approach takes the common linear modelling approach [7] for log-ratios and adjusts it so that instead of estimating terms representing relative levels of expression for the different groups we estimate terms representing absolute levels of expression. For example, instead of estimating the level for cancer sample relative to a reference, C_*R*_, and the level for a normal sample relative to a reference, N_*R*_, as would normally be the case,

(y1⋮yn)=[10⋮⋮01](CRNR)+(ε1⋮εn)     (1)
 MathType@MTEF@5@5@+=feaafiart1ev1aaatCvAUfKttLearuWrP9MDH5MBPbIqV92AaeXatLxBI9gBaebbnrfifHhDYfgasaacH8akY=wiFfYdH8Gipec8Eeeu0xXdbba9frFj0=OqFfea0dXdd9vqai=hGuQ8kuc9pgc9s8qqaq=dirpe0xb9q8qiLsFr0=vr0=vr0dc8meaabaqaciaacaGaaeqabaqabeGadaaakeaadaqadaqaauaabeqadeaaaeaacqWG5bqEdaWgaaWcbaGaeGymaedabeaaaOqaaiabl6UinbqaaiabdMha5naaBaaaleaacqWGUbGBaeqaaaaaaOGaayjkaiaawMcaaiabg2da9maadmaabaqbaeqabmGaaaqaaiabigdaXaqaaiabicdaWaqaaiabl6Uinbqaaiabl6UinbqaaiabicdaWaqaaiabigdaXaaaaiaawUfacaGLDbaadaqadaqaauaabeqaceaaaeaacqWGdbWqdaWgaaWcbaGaemOuaifabeaaaOqaaiabd6eaonaaBaaaleaacqWGsbGuaeqaaaaaaOGaayjkaiaawMcaaiabgUcaRmaabmaabaqbaeqabmqaaaqaaGGaciab=v7aLnaaBaaaleaacqaIXaqmaeqaaaGcbaGaeSO7I0eabaGae8xTdu2aaSbaaSqaaiabd6gaUbqabaaaaaGccaGLOaGaayzkaaGaaCzcaiaaxMaadaqadaqaaiabigdaXaGaayjkaiaawMcaaaaa@5552@

we instead formulate the equation in terms of absolute values (since a log-ratio is simply a linear combination of two log-expression levels) corresponding to cancer, normal and reference samples (C, N, and R respectively).

(y1⋮yn)=[10−1⋮⋮⋮01−1](CNR)+(ε1⋮εn)     (2)
 MathType@MTEF@5@5@+=feaafiart1ev1aaatCvAUfKttLearuWrP9MDH5MBPbIqV92AaeXatLxBI9gBaebbnrfifHhDYfgasaacH8akY=wiFfYdH8Gipec8Eeeu0xXdbba9frFj0=OqFfea0dXdd9vqai=hGuQ8kuc9pgc9s8qqaq=dirpe0xb9q8qiLsFr0=vr0=vr0dc8meaabaqaciaacaGaaeqabaqabeGadaaakeaadaqadaqaauaabeqadeaaaeaacqWG5bqEdaWgaaWcbaGaeGymaedabeaaaOqaaiabl6UinbqaaiabdMha5naaBaaaleaacqWGUbGBaeqaaaaaaOGaayjkaiaawMcaaiabg2da9maadmaabaqbaeqabmWaaaqaaiabigdaXaqaaiabicdaWaqaaiabgkHiTiabigdaXaqaaiabl6Uinbqaaiabl6Uinbqaaiabl6UinbqaaiabicdaWaqaaiabigdaXaqaaiabgkHiTiabigdaXaaaaiaawUfacaGLDbaadaqadaqaauaabeqadeaaaeaacqWGdbWqaeaacqWGobGtaeaacqWGsbGuaaaacaGLOaGaayzkaaGaey4kaSYaaeWaaeaafaqabeWabaaabaacciGae8xTdu2aaSbaaSqaaiabigdaXaqabaaakeaacqWIUlstaeaacqWF1oqzdaWgaaWcbaGaemOBa4gabeaaaaaakiaawIcacaGLPaaacaWLjaGaaCzcamaabmaabaGaeGOmaidacaGLOaGaayzkaaaaaa@5969@

The design matrix at this point has become singular. However this formulation allows us to insert additional rows corresponding to arrays providing only a single channel of data and thus a single log-expression value:

(y1⋮ynye1⋮yem)=[10−1⋮⋮⋮01−10−10⋮⋮⋮−100](CNR)+(ε1⋮εnε′1⋮ε′m)     (3)
 MathType@MTEF@5@5@+=feaafiart1ev1aaatCvAUfKttLearuWrP9MDH5MBPbIqV92AaeXatLxBI9gBaebbnrfifHhDYfgasaacH8akY=wiFfYdH8Gipec8Eeeu0xXdbba9frFj0=OqFfea0dXdd9vqai=hGuQ8kuc9pgc9s8qqaq=dirpe0xb9q8qiLsFr0=vr0=vr0dc8meaabaqaciaacaGaaeqabaqabeGadaaakeaadaqadaqaauaabeqageaaaaqaaiabdMha5naaBaaaleaacqaIXaqmaeqaaaGcbaGaeSO7I0eabaGaemyEaK3aaSbaaSqaaiabd6gaUbqabaaakeaacqWG5bqEdaWgaaWcbaGaemyzauMaeGymaedabeaaaOqaaiabl6UinbqaaiabdMha5naaBaaaleaacqWGLbqzcqWGTbqBaeqaaaaaaOGaayjkaiaawMcaaiabg2da9maadmaabaqbaeqabyWaaaaabaGaeGymaedabaGaeGimaadabaGaeyOeI0IaeGymaedabaGaeSO7I0eabaGaeSO7I0eabaGaeSO7I0eabaGaeGimaadabaGaeGymaedabaGaeyOeI0IaeGymaedabaGaeGimaadabaGaeyOeI0IaeGymaedabaGaeGimaadabaGaeSO7I0eabaGaeSO7I0eabaGaeSO7I0eabaGaeyOeI0IaeGymaedabaGaeGimaadabaGaeGimaadaaaGaay5waiaaw2faamaabmaabaqbaeqabmqaaaqaaiabdoeadbqaaiabd6eaobqaaiabdkfasbaaaiaawIcacaGLPaaacqGHRaWkdaqadaqaauaabeqageaaaaqaaGGaciab=v7aLnaaBaaaleaacqaIXaqmaeqaaaGcbaGaeSO7I0eabaGae8xTdu2aaSbaaSqaaiabd6gaUbqabaaakeaacuWF1oqzgaqbamaaBaaaleaacqaIXaqmaeqaaaGcbaGaeSO7I0eabaGaf8xTduMbauaadaWgaaWcbaGaemyBa0gabeaaaaaakiaawIcacaGLPaaacaWLjaGaaCzcamaabmaabaGaeG4mamdacaGLOaGaayzkaaaaaa@7911@

The design matrix is no longer singular, and the only thing that prevents the fitting of a simple linear model is that the residual errors of the log-expression measurements have a different form to those of the log-ratios. We assume that the only difference is a scaling of the variance, and that for any particular gene, this is constant across the arrays. Thus we can write the equation

(y1⋮ynye1⋮yem)=[10−1⋮⋮⋮01−10−10⋮⋮⋮−100](CNR)+(ε1⋮εn1kεn+1⋮1kεn+m)     (4)
 MathType@MTEF@5@5@+=feaafiart1ev1aaatCvAUfKttLearuWrP9MDH5MBPbIqV92AaeXatLxBI9gBaebbnrfifHhDYfgasaacH8akY=wiFfYdH8Gipec8Eeeu0xXdbba9frFj0=OqFfea0dXdd9vqai=hGuQ8kuc9pgc9s8qqaq=dirpe0xb9q8qiLsFr0=vr0=vr0dc8meaabaqaciaacaGaaeqabaqabeGadaaakeaadaqadaqaauaabeqageaaaaqaaiabdMha5naaBaaaleaacqaIXaqmaeqaaaGcbaGaeSO7I0eabaGaemyEaK3aaSbaaSqaaiabd6gaUbqabaaakeaacqWG5bqEdaWgaaWcbaGaemyzauMaeGymaedabeaaaOqaaiabl6UinbqaaiabdMha5naaBaaaleaacqWGLbqzcqWGTbqBaeqaaaaaaOGaayjkaiaawMcaaiabg2da9maadmaabaqbaeqabyWaaaaabaGaeGymaedabaGaeGimaadabaGaeyOeI0IaeGymaedabaGaeSO7I0eabaGaeSO7I0eabaGaeSO7I0eabaGaeGimaadabaGaeGymaedabaGaeyOeI0IaeGymaedabaGaeGimaadabaGaeyOeI0IaeGymaedabaGaeGimaadabaGaeSO7I0eabaGaeSO7I0eabaGaeSO7I0eabaGaeyOeI0IaeGymaedabaGaeGimaadabaGaeGimaadaaaGaay5waiaaw2faamaabmaabaqbaeqabmqaaaqaaiabdoeadbqaaiabd6eaobqaaiabdkfasbaaaiaawIcacaGLPaaacqGHRaWkdaqadaqaauaabeqageaaaaqaaGGaciab=v7aLnaaBaaaleaacqaIXaqmaeqaaaGcbaGaeSO7I0eabaGae8xTdu2aaSbaaSqaaiabd6gaUbqabaaakeaadaWcaaqaaiabigdaXaqaamaakaaabaGaem4AaSgaleqaaaaakiab=v7aLnaaBaaaleaacqWGUbGBcqGHRaWkcqaIXaqmaeqaaaGcbaGaeSO7I0eabaWaaSaaaeaacqaIXaqmaeaadaGcaaqaaiabdUgaRbWcbeaaaaGccqWF1oqzdaWgaaWcbaGaemOBa4Maey4kaSIaemyBa0gabeaaaaaakiaawIcacaGLPaaacaWLjaGaaCzcamaabmaabaGaeGinaqdacaGLOaGaayzkaaaaaa@8291@

This model could be fitted as it is, however we prefer to first estimate *k *and then fit the model for three reasons. First, the data could by chance lead to extreme estimates of *k*, and in a two stage approach we have the opportunity to adjust the value to prevent this. Second, this approach allows for easier investigation of the importance of the estimate of *k*. Finally, this approach allows us to link in to existing packages such as Limma [7] more easily.

Given we are taking this approach, the model can be rewritten once more to give

(y1⋮ynkye1⋮kyem)=[10−1⋮⋮⋮01−10−k0⋮⋮⋮−k00](CNR)+(ε1⋮εnεn+1⋮εn+m)     (5)
 MathType@MTEF@5@5@+=feaafiart1ev1aaatCvAUfKttLearuWrP9MDH5MBPbIqV92AaeXatLxBI9gBaebbnrfifHhDYfgasaacH8akY=wiFfYdH8Gipec8Eeeu0xXdbba9frFj0=OqFfea0dXdd9vqai=hGuQ8kuc9pgc9s8qqaq=dirpe0xb9q8qiLsFr0=vr0=vr0dc8meaabaqaciaacaGaaeqabaqabeGadaaakeaadaqadaqaauaabeqageaaaaqaaiabdMha5naaBaaaleaacqaIXaqmaeqaaaGcbaGaeSO7I0eabaGaemyEaK3aaSbaaSqaaiabd6gaUbqabaaakeaadaGcaaqaaiabdUgaRbWcbeaakiabdMha5naaBaaaleaacqWGLbqzcqaIXaqmaeqaaaGcbaGaeSO7I0eabaWaaOaaaeaacqWGRbWAaSqabaGccqWG5bqEdaWgaaWcbaGaemyzauMaemyBa0gabeaaaaaakiaawIcacaGLPaaacqGH9aqpdaWadaqaauaabeqagmaaaaqaaiabigdaXaqaaiabicdaWaqaaiabgkHiTiabigdaXaqaaiabl6Uinbqaaiabl6Uinbqaaiabl6UinbqaaiabicdaWaqaaiabigdaXaqaaiabgkHiTiabigdaXaqaaiabicdaWaqaaiabgkHiTmaakaaabaGaem4AaSgaleqaaaGcbaGaeGimaadabaGaeSO7I0eabaGaeSO7I0eabaGaeSO7I0eabaGaeyOeI0YaaOaaaeaacqWGRbWAaSqabaaakeaacqaIWaamaeaacqaIWaamaaaacaGLBbGaayzxaaWaaeWaaeaafaqabeWabaaabaGaem4qameabaGaemOta4eabaGaemOuaifaaaGaayjkaiaawMcaaiabgUcaRmaabmaabaqbaeqabyqaaaaabaacciGae8xTdu2aaSbaaSqaaiabigdaXaqabaaakeaacqWIUlstaeaacqWF1oqzdaWgaaWcbaGaemOBa4gabeaaaOqaaiab=v7aLnaaBaaaleaacqWGUbGBcqGHRaWkcqaIXaqmaeqaaaGcbaGaeSO7I0eabaGae8xTdu2aaSbaaSqaaiabd6gaUjabgUcaRiabd2gaTbqabaaaaaGccaGLOaGaayzkaaGaaCzcaiaaxMaadaqadaqaaiabiwda1aGaayjkaiaawMcaaaaa@81BB@

that is of a form more readily integrable into existing microarray analysis packages.

### Estimating *k*

The interpretation of *k *is as the ratio of the variance of the residuals arising from the arrays providing two channels and the variance of the residuals arising from the arrays providing only one channel. There are a number of approaches to obtaining an initial estimate of *k*, and an equally diverse range of refinements that can be applied. Our experience is that any sensible combination will lead to broadly the same results. Our approach is to take the simplest estimate of *k *by fitting a model with *k *set to be equal to one and estimating the variances of firstly those residuals associated with log-ratios and secondly those associated with log-intensities. Once 22,575 estimates of *k *are obtained (one for each spot on the array), then we use a basic shrinkage method to move those estimates inwards towards their common average. We are not employing the full empirical Bayesian shrinkage method [8], but have noticed no differences due to this, in part because the large sample sizes seen here mean that the prior distribution is not very influential in any case. Full details of the method we have used for shrinking the estimates of *k *can be found in Additional file 3.

The values of 0.25 and 0.5 were considered for use as a fixed estimate of *k *based on observations previously made by one of the authors [9] (available from [6]). There it is observed that in one experiment, contrasts formed from single channels have a standard deviation of the order of twice that for within-array log-ratios, while in another experiment the ratio was closer to three.

Assuming that the observations forming the single channel contrasts were independent and individually had variance *v*, it is easy to see that the variance of the single channel contrast would have been 2*v*, while from our definition of *k *the variance of the log-ratios would have been *kv*. From the experimental observations we have 2vkv
 MathType@MTEF@5@5@+=feaafiart1ev1aaatCvAUfKttLearuWrP9MDH5MBPbIqV92AaeXatLxBI9gBaebbnrfifHhDYfgasaacH8akY=wiFfYdH8Gipec8Eeeu0xXdbba9frFj0=OqFfea0dXdd9vqai=hGuQ8kuc9pgc9s8qqaq=dirpe0xb9q8qiLsFr0=vr0=vr0dc8meaabaqaciaacaGaaeqabaqabeGadaaakeaadaGcaaqaamaalaaabaGaeGOmaiJaemODayhabaGaem4AaSMaemODayhaaaWcbeaaaaa@3212@ = 2 or 2vkv
 MathType@MTEF@5@5@+=feaafiart1ev1aaatCvAUfKttLearuWrP9MDH5MBPbIqV92AaeXatLxBI9gBaebbnrfifHhDYfgasaacH8akY=wiFfYdH8Gipec8Eeeu0xXdbba9frFj0=OqFfea0dXdd9vqai=hGuQ8kuc9pgc9s8qqaq=dirpe0xb9q8qiLsFr0=vr0=vr0dc8meaabaqaciaacaGaaeqabaqabeGadaaakeaadaGcaaqaamaalaaabaGaeGOmaiJaemODayhabaGaem4AaSMaemODayhaaaWcbeaaaaa@3212@ = 3 resulting in estimates for *k *of 0.5 and 0.22 which for simplicity we approximate as 0.5 and 0.25.

### Normalization for the combined method

We are aiming for an object that contains M-values (log-ratios) from the unaffected arrays, and G-values (Cy3 log-intensities) from the affected arrays. The basic policy is to create an idealized set of G-values from the good arrays and normalize the bad arrays to these. We begin by performing a loess within-array normalization, although other preferences regarding the choice of within-arrays normalization can easily be accommodated. Within the good arrays, we then perform a 'scale' normalization between arrays and back calculate log-intensities for the green channel. A quantile normalization of these green log-intensities provides us our vector of idealized values. The green log-intensities are then normalized to these values by their within-array ranks.

For the production of heatmaps and clustering, this object is clearly troublesome since the arrays providing M-values will naturally cluster together apart from those supplying G-values. We can address this by back-calculating R values from the normalized unaffected arrays. Since in our design each represents the same reference sample, we take the mean red log-intensity for each spot from the unaffected arrays to get a vector of R values. From this vector we subtract the green log-intensities of the ozone-affected arrays to obtain M-values.

There is still a danger at this point of separating out the affected and unaffected arrays if performing a cluster analysis, so having obtained M-values for all arrays, a final normalization is required. For the unaffected arrays, a quantile normalization is performed, generating a vector of M-values that are seen on every array. The M-values on the ozone-affected arrays are then normalized to this vector by rank order (the highest M-value being replaced by the highest value in the vector, and so on). This leaves an object that can be successfully used for generating heatmaps or performing cluster analyses.

### Data simulation

To compare the methods available, we generated datasets using an approach inspired by that of Lonnstedt [10]. A real dataset of 55 microarrays, relating to homogenous samples, containing data for 21,073 spots was used in the generation of a dataset of 100 microarrays containing data for 22,575 spots. These 100 were divided into two groups, one of size 70 and the other of size 30. 4,000 of the simulated spots were differentially expressed between the two groups.

From the real data we have a matrix of log-ratios *M*_*ag*_ and a corresponding matrix of log-intensities for the red channel *R*_*ag *_where 1 ≤ *a *≤ 55, and 1 ≤ *g *≤ 21,073. From this we create a vector of the average log-ratios associated with the genes, *avM*, where *avM*_*g *_= 155
 MathType@MTEF@5@5@+=feaafiart1ev1aaatCvAUfKttLearuWrP9MDH5MBPbIqV92AaeXatLxBI9gBaebbnrfifHhDYfgasaacH8akY=wiFfYdH8Gipec8Eeeu0xXdbba9frFj0=OqFfea0dXdd9vqai=hGuQ8kuc9pgc9s8qqaq=dirpe0xb9q8qiLsFr0=vr0=vr0dc8meaabaqaciaacaGaaeqabaqabeGadaaakeaadaWcaaqaaiabigdaXaqaaiabiwda1iabiwda1aaaaaa@2F9C@ ∑_*a *_*M*_*ag*_, and a matrix, Δ, of the differences between log-ratios and their gene average, where Δ_*ag *_= *M*_*ag *_- *avM*_*g*_.

A new matrix of log-ratios, *M'*, is then built row-by-row, where M′b,h
 MathType@MTEF@5@5@+=feaafiart1ev1aaatCvAUfKttLearuWrP9MDH5MBPbIqV92AaeXatLxBI9gBaebbnrfifHhDYfgasaacH8akY=wiFfYdH8Gipec8Eeeu0xXdbba9frFj0=OqFfea0dXdd9vqai=hGuQ8kuc9pgc9s8qqaq=dirpe0xb9q8qiLsFr0=vr0=vr0dc8meaabaqaciaacaGaaeqabaqabeGadaaakeaacuWGnbqtgaqbamaaBaaaleaacqWGIbGycqGGSaalcqWGObaAaeqaaaaa@318D@ = *avM*_*x*(*h*) _+ Δ_*g*(*b*),*f*(*x*(*h*)) _+ ε. Here *x*() associates each row of *M' *with an element of *avM *at random, *g*() represents a random sample (with replacement) of 100 values from 1 to 55, and *f*() returns a row of Δ that is in some sense near the row associated with the element of *avM *that was returned by *x*(). ε adds a small amount of noise into the mix to avoid identical elements appearing. Here 1 ≤ *b *≤ 100, and 1 ≤ *h *≤ 22,575.

A new matrix of corresponding log-intensities, *R'*, for the red channel is then constructed as R′b,h
 MathType@MTEF@5@5@+=feaafiart1ev1aaatCvAUfKttLearuWrP9MDH5MBPbIqV92AaeXatLxBI9gBaebbnrfifHhDYfgasaacH8akY=wiFfYdH8Gipec8Eeeu0xXdbba9frFj0=OqFfea0dXdd9vqai=hGuQ8kuc9pgc9s8qqaq=dirpe0xb9q8qiLsFr0=vr0=vr0dc8meaabaqaciaacaGaaeqabaqabeGadaaakeaacuWGsbGugaqbamaaBaaaleaacqWGIbGycqGGSaalcqWGObaAaeqaaaaa@3197@ = *R*_*g*(*b*),*f*(*x*(*h*))_, where *g*,*f*, and *x *are defined as above. For 4000 of the simulated log-ratios, in 30 of the simulated arrays, some differential expression was introduced by adding an extra term into the formula for M′b,h
 MathType@MTEF@5@5@+=feaafiart1ev1aaatCvAUfKttLearuWrP9MDH5MBPbIqV92AaeXatLxBI9gBaebbnrfifHhDYfgasaacH8akY=wiFfYdH8Gipec8Eeeu0xXdbba9frFj0=OqFfea0dXdd9vqai=hGuQ8kuc9pgc9s8qqaq=dirpe0xb9q8qiLsFr0=vr0=vr0dc8meaabaqaciaacaGaaeqabaqabeGadaaakeaacuWGnbqtgaqbamaaBaaaleaacqWGIbGycqGGSaalcqWGObaAaeqaaaaa@318D@. After examination of the results of models fitted to the real data, the differential expression was simulated as being from a mixture of three normal distributions, with means at -0.9, 0, and 0.9, variances of 0.25, 1, and 0.25 and weights of 0.4375, 0.125, and 0.4375 respectively.

Green log intensities were then back-calculated from the red log-intensities and the log-ratios. The green and red values were used as the basis of the analysis. Where appropriate, the red log-intensities were damaged as follows: From the real data, matrices of log-intensities, *RD *and *GD*, from damaged microarrays and *RU *and *GU *from undamaged arrays were constructed using only those microarrays that compared a cancer sample to the reference and with some undamaged microarrays omitted so as to create matrices of equal size.

A matrix of predicted red log-intensities (for the case had there been no damage) was produced for the damaged arrays as *PD*_*ij *_= *RU*_*ij*_*GD*_*ij*_/*GU*_*ij *_and from this, an ozone damage matrix, *OD*, was produced such that *OD*_*ij *_= *RD*_*ij*_/*PD*_*ij*_. Damage to the red intensities was then simulated by multiplying log-intensities by values sampled from *OD*.

### The simulation experiment

One hundred datasets were simulated in the specified manner, consisting of four groups of 25 datasets, each group with the red channel corrupted for a different proportion of the arrays. The damage levels simulated were such that either 20, 40, 60 or 80 of the 100 arrays in a dataset would have their red channel corrupted. Six analyses were then performed on each simulated dataset. These were an analysis of only the single green channel, an analysis of all of the data (ignoring the problem entirely), an analysis of those arrays that did not experience problems with the red channel, and three analyses as presented in this paper (one actively estimating *k *as described above, one with *k *prespecified to be 0.25 and another with the value set at 0.5).

• "Single channel analysis": Quantile normalization of the 'green' channel followed by linear modelling.

• "Inclusive analysis": For all of the data, regardless of the damage to the 'red' channel, construction of log-ratios, within-array loess normalization to the average intensities, linear modelling of the log-ratios.

• "Unaffected data" analysis: As for the 'Inclusive analysis', but only for those arrays with unaffected 'red' channels.

• The "Combined(*k*_est_)" approach: as presented in this article, using *k *estimated from the data.

• The "Combined(*k*_fix_)" approach: as presented in this article, but with *k *set equal to 0.25.

• The "Combined(*k*_fix_)" approach: as presented in this article, but with *k *set equal to 0.5.

### Analysing data from the simulation experiment

Methods were primarily assessed by their sensitivity and specificity for all lengths of genelist. Additionally, since 2,000 genes were known to be differentially expressed, we also looked specifically at gene-lists of length 2,000. Basic comparisons were via percentages of differentially expressed genes correctly returned, which were then broken down both by proportion of missing channels and size of differential effect. A more powerful comparison of any two methods is possible by a McNemar-type test (actually the exact binomial equivalent). All such paired comparisons are conducted in a pairwise manner. No contrary results (i.e. *A *> *B*, *B *> *C*, *C *> *A*) were seen.

### Software

Analyses were conducted using R 2.1.1 [11], and the Limma package [7].

## Authors' contributions

DEN, JDK and GJB obtained the samples and produced the motivating data. GJB and NPT identified the issue of damage to the red channel. NPT conceived of the possibility of an improved analysis, oversaw the research and helped to draft the manuscript, AGL developed and performed the analyses, and drafted the manuscript. All authors read and approved the final manuscript.

## Supplementary Material

Additional File 1 Presented are the results of the simulation experiment. This file has six pages corresponding to 1) A key to the sheets presenting paired comparisons. 2) Paired comparisons of the methods as a whole, and stratified by the percentage of arrays that are damaged. 3) Unpaired comparisons (including reproduction of Table 2) as a whole, and stratified by the size of the differential expression effects. 4) As for page 2, but only for differential expressions that were < 0.5 in magnitude. 5) As for page 2, but only for differential expressions that were ≥ 0.5 and < 1 in magnitude. 6) As for page 2, but only for differential expressions that were ≥ 1 in magnitude. Click here for file

Additional File 2 Lists of the rankings attributed to the top genes by the different methods are presented in an Excel file. Click here for file

Additional File 3 Details of the approach we took to estimating the values of *k*. Click here for file
