# Supplementary material for: Missing channels in two-colour microarray experiments: Combining single-channel and two-channel data
Source: BMC Bioinformatics. 2007 Jan 25;8:26. doi: 10.1186/1471-2105-8-26 (PMC1797192; doi:10.1186/1471-2105-8-26)
Supplement: Additional File 3 — Details of the approach we took to estimating the values of k. [file 1471-2105-8-26-S3.pdf]

The Shrinkage Step

This document gives details of the shrinkage step used in the process of estimating the parameter k, the ratio of variances associated with a log-intensity and a ratio of log-intensities. Our manuscript concerns the principle of a combined analysis and indeed shows that the active estimation of k may not even be necessary to gain sensible results. Thus we can assume that the exact nature of the shrinkage applied is not critical. The complexity of the shrinkage step is disproportionate to its importance and is thus relegated to this document.

Rather than using our observation $\hat{k}$ of the estimator $\overline{k}$ for the value *k*, we would prefer to replace it with an estimate of $E\left(\overline{k}\right)$, adjusted for prior beliefs about the form of *k*.

The Likelihood

If we have independent estimates of the variance from the *n* residuals associated with the log-ratios (S_R^2) and from the *m* residuals associated with log-intensities (S_E^2) from the affected arrays then we know

$$\overline{k} \sim kF_{a,u} \tag{1.1}$$

$$E_F = E\left(\overline{k}\right) = k\frac{u}{u-2} \tag{1.2}$$

$$V_F = Var(\overline{k}) = k^2\frac{2u^2\left(a+u-2\right)}{a\left(u-2\right)^2\left(u-4\right)} \qquad (1.3)(1.4)$$

Where a (affected) = $m - 1$ and u (unaffected) = $n - 1$

For computational ease we will approximate the distribution of $\bar{k}$ using a log-normal distribution.

The expectation of a log-normal$\left(\mu, \tau^2\right)$ distribution is

$$E_{\text{LN}} = \exp\left(\mu + \frac{\tau^2}{2}\right). \quad (1.4)$$

While the variance is

$$V_{\text{LN}} = \exp\left(2\mu + 2\tau^2\right) - \exp\left(2\mu + \tau^2\right). \quad (1.5)$$

$$V_{\text{LN}} = \exp\left(2\mu + 2\tau^2\right) - E_{\text{LN}}{}^2 \quad (1.6)$$

We can rearrange these to get expressions for mu and sigma.

$$(1.4) \Rightarrow \quad 2\mu + \tau^2 = \log\left(E_{\text{LN}}{}^2\right) \quad (1.7)$$

$$(1.6) \Rightarrow \quad 2\mu + 2\tau^2 = \log\left(V_{\text{LN}} + E_{\text{LN}}{}^2\right) \quad (1.8)$$

$$(1.7),(1.8) \Rightarrow \quad \tau^2 = \log\left(V_{\text{LN}} + E_{\text{LN}}{}^2\right) - \log\left(E_{\text{LN}}{}^2\right) = \log\left(\frac{V_{\text{LN}} + E_{\text{LN}}{}^2}{E_{\text{LN}}{}^2}\right) \quad (1.9)$$

$$(1.7),(1.9) \Rightarrow \quad 2\mu = \log\left(E_{\text{LN}}{}^2\right) - \log\left(\frac{V_{\text{LN}} + E_{\text{LN}}{}^2}{E_{\text{LN}}{}^2}\right) = \log\left(\frac{E_{\text{LN}}{}^4}{V_{\text{LN}} + E_{\text{LN}}{}^2}\right)$$

$$\mu = \log\left(\frac{E_{\text{LN}}{}^2}{\sqrt{V_{\text{LN}} + E_{\text{LN}}{}^2}}\right) \quad (1.10)$$

We approximate the F distribution with a log-normal distribution by equating moments. So $\bar{k}$ has a log-normal$\left(\mu, \tau^2\right)$ distribution where

$$\mu = \log\left[\frac{E_F^2}{\sqrt{V_F + E_F^2}}\right] \tag{1.11}$$

and
$$\tau^2 = \log\left[\frac{V_F + E_F^2}{E_F^2}\right]. \tag{1.12}$$

So $\theta = \log\left(\bar{k}\right) \sim N\left(\mu, \tau^2\right)$ where mu and tau are defined as above.

(1.11),(1.2),(1.3) $\Rightarrow$
$$\exp\left(\mu\right) = k\frac{u}{u-2}\sqrt{\frac{\left(u-4\right)a}{2\left(a+u-2\right)^2 + \left(u-4\right)a}} \tag{1.13}$$

(1.12),(1.2),(1.3) $\Rightarrow$
$$\exp\left(\tau^2\right) = \frac{2\left(a+u-2\right)^2}{a\left(u-4\right)} \tag{1.14}$$

Since k is unknown we substitute our observation $\hat{k}$ into (1.13) to obtain

$$\exp\left(\mu\right) = \frac{s_E^2}{s_R^2}\frac{u}{u-2}\sqrt{\frac{\left(u-4\right)a}{2\left(a+u-2\right)^2 + \left(u-4\right)a}} \tag{1.15}$$

The Prior

We suppose that we have a log-normal prior distribution for $\bar{k}$, $\theta = \log\left(\bar{k}\right) \sim N\left(a, b^2\right)$.

The Posterior

It is then a standard result that we can formulate the posterior distribution of θ as $\theta \sim N\left(\mu_p, \sigma_p^2\right)$ where

$$\omega = \frac{\tau^2}{\tau^2 + b^2} \tag{1.16}$$

$$\sigma_p^2 = \frac{\tau^2 b^2}{\tau^2 + b^2} \tag{1.17}$$

$$\mu_p = \omega a + (1-\omega)\,\mu \tag{1.18}$$

From (1.4) the posterior expected value of $\bar{k}$ is therefore

$$E\left(\bar{k} \mid \hat{k}, a, b\right) = \exp\left(\mu_p + \frac{\sigma_p^2}{2}\right) \tag{1.19}$$

Which is the value we use.

Choice of Prior

Where we have compromised is in the estimates of a and b^2 that we use. For simplicity we look at the empirical distribution the 22575 observations of $\bar{k}$ to provide direct estimates a and b^2; ignoring the contribution of τ^2 to the observed variance. Due to the small magnitude of τ^2 relative to b^2, this compromise has negligible effect in our example. Indeed the shrinkage generally only has a small effect due to the large sample size.

AGL 2006
